# A Stable Dried Tube Specimen for Quality Assurance and Training Programs for HIV Rapid Test for Recent Infection

**DOI:** 10.1128/spectrum.03398-22

**Published:** 2023-01-17

**Authors:** Clara Di Germanio, Ernest L. Yufenyuy, Dylan C. Hampton, Chloe Thorbrogger, Bharat S. Parekh, Philip J. Norris

**Affiliations:** a Vitalant Research Institute, San Francisco, California, USA; b Department of Laboratory Medicine, University of California, San Francisco, San Francisco, California, USA; c Division of Global HIV and TB, Center for Global Health, Centers for Disease Control and Prevention, Atlanta, Georgia, USA; National Institute of Allergy and Infectious Diseases

**Keywords:** HIV rapid test, HIV recency infection, human immunodeficiency virus

## Abstract

The HIV epidemic is still one of the world’s most serious public health challenges, affecting about 38 million people worldwide, especially in sub-Saharan African and Southeast Asian countries. In recent years, tests have been developed to discriminate recent from long-term infection in HIV-infected populations, and these tools can help identify new outbreaks and networks of transmission and target prevention and treatment plans. New rapid tests for recent infection are being deployed in point-of-care settings; however, quality assurance programs need to be implemented to ensure consistency and reliability of the results. We have developed a dried tube specimen (DTS) stabilized with disaccharide trehalose as a quality control reagent for rapid recency testing that can be stored unrefrigerated prior to reconstitution at temperatures up to 37°C for up to 12 weeks. Analysis of 10 trehalose-stabilized DTSs showed that they maintained the same recency classification in all of the samples stored at 4°C and 37°C up to 12 weeks and at 56°C for 2 weeks, while the DTSs prepared without trehalose changed their classification from long-term to recent or recent to negative after storage at 37°C for 12 weeks. Development of DTS quality control reagents will facilitate proficiency and training programs, particularly in settings without cold chain capability in field environments.

**IMPORTANCE** Implementation of stabilized dried tube specimens (DTSs) for quality control and training would facilitate HIV recency programs, especially in point-of-care settings without cold chain availability. This study shows that addition of the disaccharide trehalose to DTSs prior to drying the samples increased stability of the samples across a range of temperatures. This finding provides an affordable way to increase the availability of these key reagents for quality control in resource-constrained settings.

## INTRODUCTION

Thirty-eight years after the identification of HIV as the causative agent of AIDS, significant progress has been made in the understanding of the virus biology and consequent disease control, but except for rare cases, a cure does not yet exist (1–4). By the end of 2020, roughly 40 million people were reported to be living with HIV globally, with almost 2 million new cases occurring annually. Geographically, more than half of the HIV-positive population lives in Eastern and Southern Africa, and this proportion rises to two-thirds of the global population if Western and Central Africa are included (5). Although pharmacological treatments exist today to control the HIV viral load and avoid further progression of the disease and spread of the virus, these drugs are required lifelong, and a segment of the population is still unaware of their HIV-positive status. Consequently, new prevention tools are needed to contain the HIV epidemic, and improved diagnosis with consequent access to treatment is one of them.

Multiple tests and algorithms exist to diagnose and stage HIV infection, and they are based on the virus nucleic acid levels, HIV antigen detection, the host antibody response, or combinations of these parameters (6). In recent years, new assays targeting early markers of HIV infection that predict recency of infection have been developed (7). These recency assays are aimed at refining estimates of HIV infection at the population level and improving early diagnosis and early treatment at the individual level (8). In fact, recently infected individuals are highly infectious due to the higher HIV concentration in the body fluids, lack of circulating neutralizing antibodies, and possibly being unaware of their status. Subjects with primary HIV infection can potentially spread the virus through risky behaviors more easily because they are unaware of their HIV-positive status compared to people who are long-term infected, particularly those with known HIV status and on antiretroviral therapy (9).

HIV rapid recency tests appear promising for point-of-care (POC) settings for recent infection surveillance to detect new HIV infections and identify transmission hot spots (10). These lateral flow tests are composed of a single-use test strip with a limiting amount of multisubtype recombinant gp41 antigen that allows differentiation of long-term from recent infection based on the avidity of the HIV antibodies, whereas low-avidity, recent antibodies do not bind as much as high-avidity, long-term antibodies present at higher concentrations (11). The human specimen is diluted in preformulated sample buffer and adsorbed onto the membrane; results are available in as little as 20 min and visually interpreted based on the appearance of long-term (LT), verification (VER), and control (CTR) lines in the results region of the strip. In this study, we utilized the Sedia Asanté assay (12).

Regardless of the choice of assay manufacturer, rapid HIV recency tests require trained personnel in order to be correctly implemented and interpreted. In addition, external quality assessment programs are a fundamental component of the laboratory quality management system and are aimed at benchmarking results from a given laboratory to ensure consistent performance and reproducibility. For this purpose, training and proficiency panels of samples are used in POC settings (13). Usually, these panels are composed of frozen plasma or serum of known HIV status; however, shipment and cold chain storage and distribution are often logistically challenging in resource-limited settings, where these interventions are needed the most.

Building on previously published techniques (14), we have developed a modified dried tube specimen (DTS) for rapid recency assays that is stable for a prolonged period at high temperature and can be reconstituted at POC without losing its test characteristics. This will help in developing and implementing POC training and quality assurance programs and improve diagnosis and recent infection surveillance in resource-limited settings.

## RESULTS

### Stability of the DTS over time and a range of temperatures.

**FIG 1:**
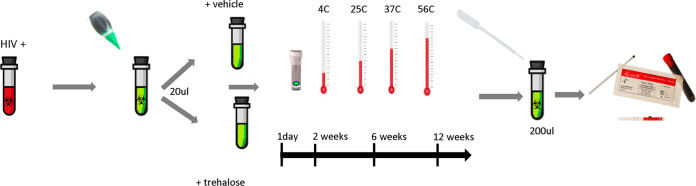
Workflow of DTS preparation. HIV-positive plasma samples were selected, then green food coloring was added (0.1%) to samples, and 20 μL of each was dried in a 2-mL tube, with or without 250 mM trehalose. Samples were stored for up to 12 weeks at different temperatures (4°C, 25°C, 37°C, and 54°C). At each time point (1 day, 2 weeks, 6 weeks, and 12 weeks), one aliquot of each sample was reconstituted in 200 μL PBS-Tween overnight and tested with the Sedia Asanté rapid recency assay kit. Numerical results were obtained with a reader provided by the manufacturer.

Samples received from Creative Testing Solutions (CTS) and the U.S. Centers for Disease Control and Prevention (CDC) were used to build the study panel to assess stability over time at different temperatures and included 4 clear LT, 4 clear recent (R), and 2 negative samples. Samples were heat inactivated and prepared into DTS with or without 250mM trehalose as preservative, and stored at different temperatures for up to 12 weeks before being reconstituted for testing (Fig. 1). Heat inactivation did not affect the sample classification for any sample (see Fig. S1 in the supplemental material).

**FIG 2:**
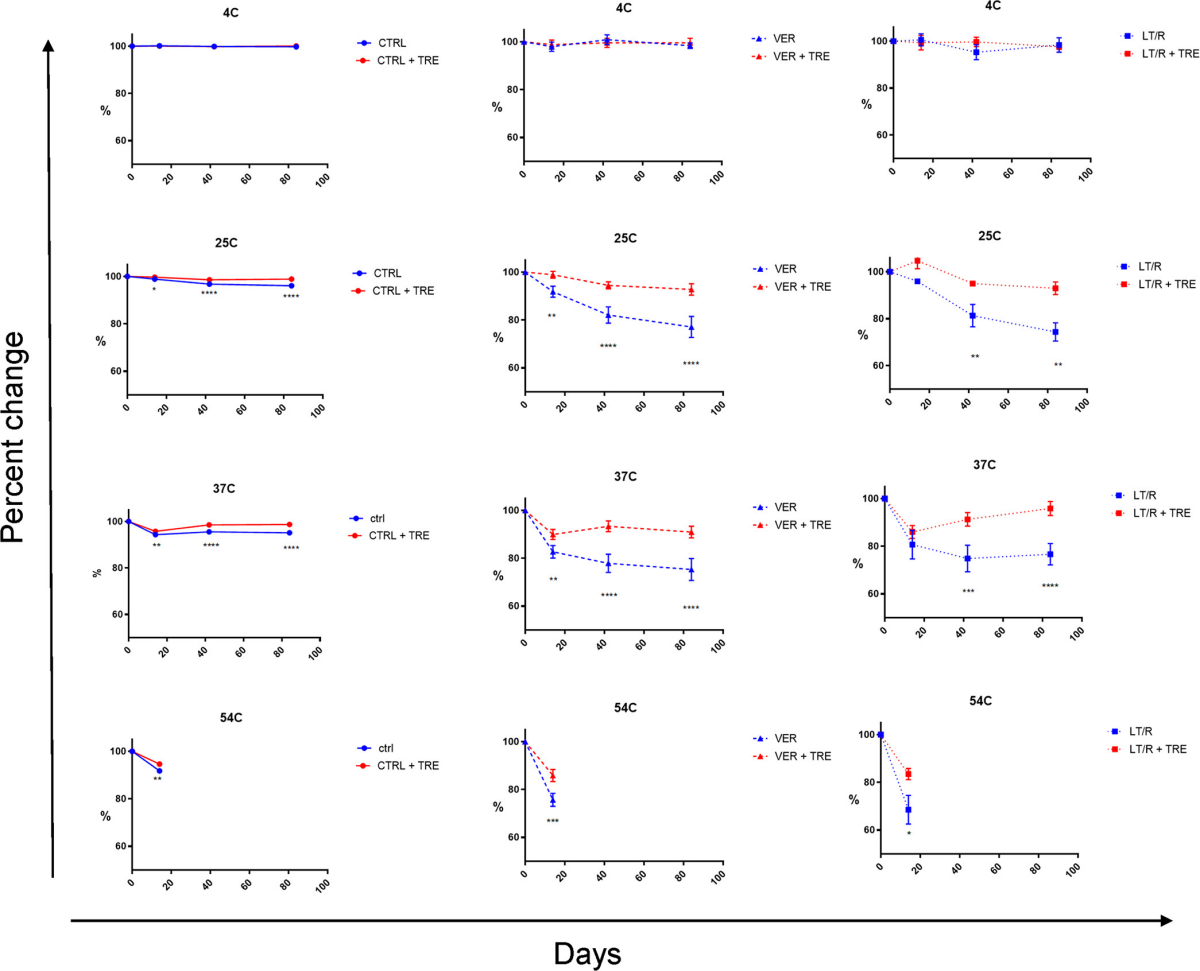
Stability of the DTS over time at different temperatures. Each line represents the average control, verification, or LT/R reading percent variation from the baseline through 12 weeks at the noted temperatures. The red lines represent the DTS samples prepared with PBS–0.1% Tween plus 250 mM trehalose, and the blue lines represent samples prepared with PBS–0.1% Tween. There were 10 samples, including 4 long-term, 4 recent, and 2 negative. Error bars represent standard errors of the means. ***, *P* < 0.001 for PBS–0.1% Tween versus PBS–0.1% Tween plus 250 mM trehalose conditions. Data were compared by ANOVA with Tukey’s posttest.

All the samples were stable at 4°C for the maximum length of the study (12 weeks) (Fig. 2). At 25°C and 37°C, the CTR line intensity was slightly but significantly higher in the trehalose-treated samples than in the controls, especially at 6 and 12 weeks (+2.8% at 25°C and +3.6% at 37°C). The VER and LT/R lines declined overall over time, but the VER line was on average 15.6% higher for the trehalose-treated DTS compared to the controls, and the LT/R line was 18.9% higher (Fig. 2).

**FIG 3:**
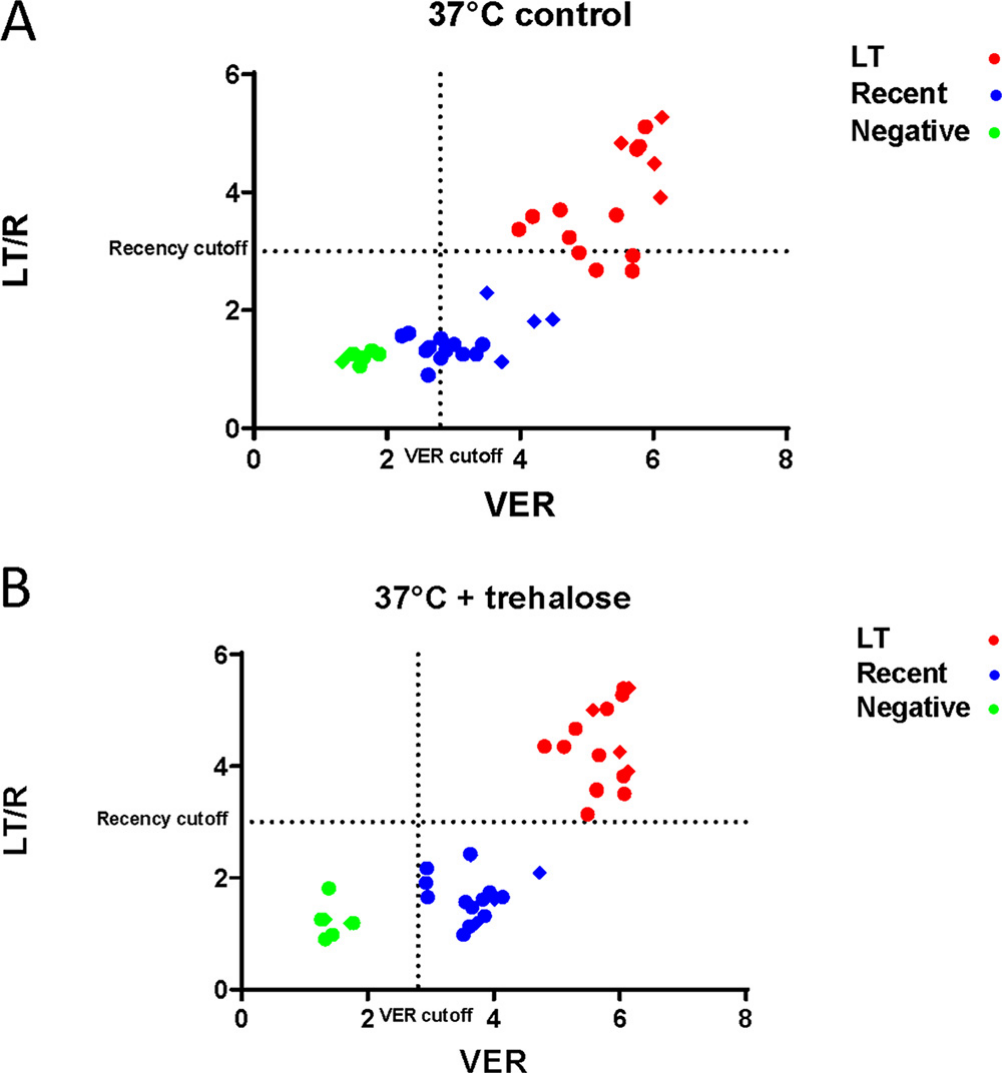
Shift in Asanté assay classification in DTSs stored at 37°C. The panel of 10 DTSs was prepared with (A) or without (B) trehalose (TRE); reader values were plotted as LT/R line versus VER. Samples that shifted classification after storage can be seen, such as blue symbols from samples classified as R in panel A that migrated to the lower left (negative) quadrant. Diamonds represent baseline values, and circles represent data for any subsequent time point (2, 6, and 12 weeks of DTS storage) at 37°C.

**FIG 4:**
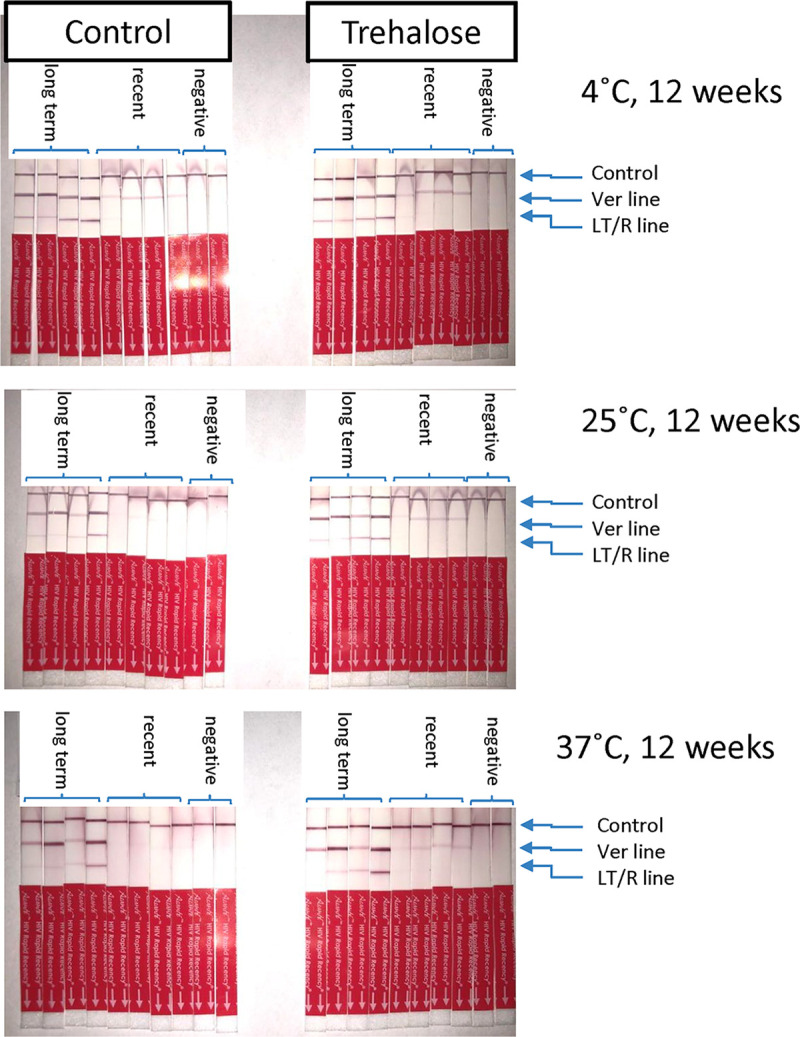
Test strips with and without trehalose. Ten representative test strips each are shown for paired samples stored at 4°C, 25°C, or 37°C for 12 weeks. CTR, control line; VER, positive verification line; LT/R, long-term/recent line.

Examination of the classification of the samples by the Asanté assay was possible by comparing the LT/R and VER lines. None of the trehalose-treated samples changed classification even at the most extreme condition (Fig. 3); all LT samples remained LT after 12 weeks at 37°C (Fig. 3B, upper right quadrant), and all recent samples stayed as recent after 12 weeks at 37°C (Fig. 3B, lower left quadrant), while multiple control DTSs changed classification—4 long-term became recent (moved from upper right quadrant to lower right) and 5 recent became negative (moved from lower right quadrant to lower left) (Fig. 3A). These results are also shown as images of developed strips in Fig. 4.

## DISCUSSION

Implementation of HIV recency testing can allow location of transmission hot spots and targeting of resources to those areas. Implementation of these assays, however, requires training the testing personnel and ongoing quality assurance to ensure that results are reliable and consistent over time at each testing site. While there are ongoing efforts to implement rapid recency testing in multiple African and Asian countries at POC (https://trace-recency.org) (8), training and external quality assurance programs currently rely on the use of samples that necessitate a difficult-to-maintain cold chain for shipping and storage of standardized panels across rural or remote areas, with a risk of degradation over time. Furthermore, identifying recently infected persons and collecting large sample volumes occurs less frequently than from those with long-term infection; thus, it is more difficult to obtain large volumes for panel preparation. Previous studies tested the stability of DTS for HIV detection for up to 4 weeks; however, those studies focused on HIV-1 serology assays (lateral flow and enzyme immunoassay formats) and quantitative nucleic acid techniques (15, 16). Standard diagnostic HIV tests are designed to be very sensitive and can detect even low levels of HIV antibodies, and one would expect that a limiting antigen assay might be more affected by degradation of antibodies stored under suboptimal conditions. In this study, we generated a DTS with trehalose as preservative for HIV recency testing and it appeared to be stable for 3 months at temperatures ranging from 4°C to 37°C.

First, it appeared that DTSs tended to be mostly stable at 4°C for up to 12 weeks without changing classification, with or without trehalose. However, our data clearly demonstrated that DTSs prepared with 250 mM trehalose were stable for up to 12 weeks at temperatures ranging from 4°C to 37°C, but antibody denaturation was rapid without trehalose. Trehalose is a disaccharide composed of two molecules of glucose (α-d-glucopyranosyl α-d-glucopyranoside dihydrate) and is naturally produced in a large number of organisms in response to environmental stress, including drought (17). In fact, it appears to have a pivotal role in stabilizing membranes and proteins during desiccation through different mechanisms, including water replacement by forming hydrogen bonds with the molecules’ residues, water entrapment by concentrating residual water at the molecule surface, and vitrification (18). Our data showed that trehalose stabilized HIV antibodies without interfering with the Asanté test, thus making it possible to ship and store samples at ambient temperature, overcoming most of the limitations, costs, and logistics that cold chains impose.

In comparison with the untreated samples, trehalose appeared to have a stronger protective effect on the control and verification line signal, more than for the LT/R line. It was unclear why, but it is possible that the limiting antigen on the LT/R line is able to detect subtle changes in HIV antibodies that occur during storage, even in the presence of trehalose.

Although this study has a few limitations, i.e., it included a small panel of samples, required an extra dilution step at the testing site, and implies an off-label use of the assay, it offers an alternative for implementing routine quality control and external quality assurance programs for rapid recency testing in resource-limited settings that would otherwise be challenging with the current protocols.

## MATERIALS AND METHODS

### Human plasma.

HIV-1-positive and -negative confirmed plasma samples for panel building were obtained from blood donors from Creative Testing Solutions (CTS; Phoenix, AZ, USA). All the samples were previously tested for HIV nucleic acid (NAT) with the Procleix Ultrio Plus on the Tigris system (Gen-Probe–Hologic, USA) and for antibodies with Abbott Prism HIV O Plus (Abbott Laboratories, USA) or the genetic system of the HIV-1/HIV-2 Plus O assay (Bio-Rad, USA). Samples were tested at Vitalant Research Institute with the Asanté HIV-1 rapid recency assay (Sedia Biosciences Corp., USA).

All samples were characterized on the Asanté HIV-1 rapid recency assay before and after heat inactivation at 56°C for 30 min to confirm the assay would not affect the sample classification. Of the 41 samples used for this evaluation, 1 was negative, 8 were recent, 3 were near the cutoff between recent and long-term (LT), and 29 were LT.

### DTS panel.

A panel of 10 representative samples was created to include 4 long-term, 4 recent, and 2 HIV-1-negative specimens. Seven samples were sourced from CTS and three from the CDC Division of Global HIV and TB, including one recent, one long-term, and one negative sample.

Samples were virus inactivated at 56°C for 30 min, and green food coloring was added at a concentration of 0.1% to make the dried samples visible. To compare sample stability over time and a range of temperatures, vehicle (phosphate-buffered saline [PBS]–0.1% Tween) or PBS–0.1% Tween with 250 mM trehalose, a disaccharide known for its ability to stabilize proteins under freezing and drying conditions, was used. Fifty microliters of 1 M trehalose in PBS–0.1% Tween stock was added to 150 μL of plasma or serum, to achieve a final concentration of 250 mM trehalose. Addition of 50 μL PBS–0.1%, without trehalose, served as a control. Addition of PBS-Tween with or without trehalose did not change the recency status of the specimen. Next, 20 μL of liquid plasma, with or without trehalose, was aliquoted into individual tubes, dried at room temperature in a biosafety cabinet overnight, and stored for 1 day, 2 weeks, 6 weeks, or 12 weeks at 4°C, 25°C, 37°C, or 54°C (Fig. 1). Specimens were then reconstituted with PBS–0.1% Tween (200 μL) before testing.

### HIV recency testing.

The Asanté HIV-1 rapid recency assay (Sedia Biosciences), a rapid lateral flow test, was performed following the manufacturer’s guidelines, except that a reconstituted DTS specimen was used when appropriate. A collection loop designed to contain 5 μL was dipped into the neat plasma or reconstituted DTS specimens and transferred into the sample buffer tube and mixed. A test strip was inserted in the tube, and after 20 min it was read on the Asanté rapid test strip reader (Sedia Biosciences, USA), with results for each line yielding a value between 0 and 7, based on the intensity of the band detected. As an alternative to eye reads, an electronic reader is available from the manufacturer (14). Appearance of the control (CTR) line, coated with goat anti-human antibodies, established the validity of the test, i.e., the presence of adequate IgG in the sample; the subsequent appearance of the verification (VER) line demonstrated the presence of HIV antibodies, while the appearance of the long-term/recent (LT/R) line indicated a long-term infection. A reading of 3.0 was the threshold for presence of the CTR and LT/R lines, and a reading of 2.8 was the threshold for the VER line.

### Statistical analysis.

Data analysis was performed using Prism 7 (GraphPad). Paired samples were analyzed using a *t* test. Multiple comparisons were analyzed by two-way analysis of variance (ANOVA) followed by an appropriate posttest. Data are expressed as means, with *P* values of ≤0.05 considered statistically significant.
