# Supplementary material for: A Stable Dried Tube Specimen for Quality Assurance and Training Programs for HIV Rapid Test for Recent Infection
Source: Microbiol Spectr. 2023 Jan 17;11(1):e03398-22. doi: 10.1128/spectrum.03398-22 (PMC9927143; doi:10.1128/spectrum.03398-22)
Supplement: Supplemental file 1 — Fig. S1. Download spectrum.03398-22-s0001.pdf, PDF file, 0.1 MB [file spectrum.03398-22-s0001.pdf]

Supp. Fig. 1

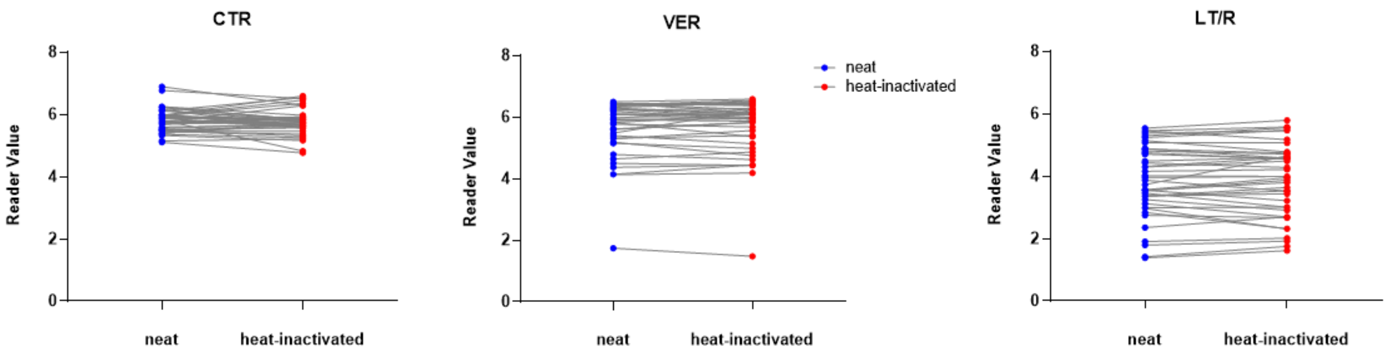

Supplementary Fig 1. Assay performance before and after heat inactivation of DTS samples.

Aliquots from 41 samples were heat inactivated at 56°C for 30 minutes. The pre-and post-heat inactivation samples showed no significant difference in signal on the CTR, VER, or LT/R lines. Comparisons were made by paired T-test.
